# Phototrophic Fe(II)-oxidation in the chemocline of a ferruginous meromictic lake

**DOI:** 10.3389/fmicb.2014.00713

**Published:** 2014-12-08

**Authors:** Xavier A. Walter, Antonio Picazo, Maria R. Miracle, Eduardo Vicente, Antonio Camacho, Michel Aragno, Jakob Zopfi

**Affiliations:** ^1^Laboratory of Microbiology, Institute of Biology, University of NeuchâtelNeuchâtel, Switzerland; ^2^Faculty of Environment and Technology, Bristol BioEnergy Centre, University of the West of EnglandBristol, UK; ^3^Department of Microbiology and Ecology, Cavanilles Institute of Biodiversity and Evolutionary Biology, University of ValenciaBurjassot, Spain; ^4^Aquatic and Stable Isotope Biogeochemistry, Department of Environmental Sciences, University of BaselBasel, Switzerland

**Keywords:** geomicrobiology, anoxygenic photosynthesis, early life evolution, banded iron formation, cryptic sulfur cycling

## Abstract

Precambrian Banded Iron Formation (BIF) deposition was conventionally attributed to the precipitation of iron-oxides resulting from the abiotic reaction of ferrous iron (Fe(II)) with photosynthetically produced oxygen. Earliest traces of oxygen date from 2.7 Ga, thus raising questions as to what may have caused BIF precipitation before oxygenic photosynthesis evolved. The discovery of anoxygenic phototrophic bacteria thriving through the oxidation of Fe(II) has provided support for a biological origin for some BIFs, but despite reports suggesting that anoxygenic phototrophs may oxidize Fe(II) in the environment, a model ecosystem of an ancient ocean where they are demonstrably active was lacking. Here we show that anoxygenic phototrophic bacteria contribute to Fe(II) oxidation in the water column of the ferruginous sulfate-poor, meromictic lake La Cruz (Spain). We observed *in-situ* photoferrotrophic activity through stimulation of phototrophic carbon uptake in the presence of Fe(II), and determined light-dependent Fe(II)-oxidation by the natural chemocline microbiota. Moreover, a photoferrotrophic bacterium most closely related to *Chlorobium ferrooxidans* was enriched from the ferruginous water column. Our study for the first time demonstrates a direct link between anoxygenic photoferrotrophy and the anoxic precipitation of Fe(III)-oxides in a ferruginous water column, providing a plausible mechanism for the bacterial origin of BIFs before the advent of free oxygen. However, photoferrotrophs represent only a minor fraction of the anoxygenic phototrophic community with the majority apparently thriving by sulfur cycling, despite the very low sulfur content in the ferruginous chemocline of Lake La Cruz.

## Introduction

The chemistry of the anoxic Archean ocean was characterized by a low sulfate content and high concentrations of ferrous iron (Fe(II)) of probable hydrothermal origin (Holland, 1973; Anbar and Knoll, 2002; Canfield, 2005). From this ferruginous water column, alternating sedimentary deposits of iron oxide minerals and silica precipitated between 3.8 and 1.8 Ga ago (Anbar and Knoll, 2002) and became preserved in the geological record as Banded Iron Formations (BIF). The mechanisms of Fe(II) oxidation are still debated and include, in addition to the widely accepted abiotic reaction with photosynthetically produced oxygen (Cloud, 1968), photocatalytic oxidation by UV radiation (Braterman et al., 1983), and direct oxidation by anoxygenic photosynthesis (Konhauser et al., 2002; Kappler et al., 2005). Such photoferrotrophic bacteria use light as energy and Fe(II) as an electron source for carbon fixation and biomass formation (Widdel et al., 1993; Heising et al., 1999) (Equation 1, Table 1).

**Table 1:** **Simplified stoichiometries of photoferrotrophic primary production (Equation 1) and different anaerobic modes of organic matter degradation (Equation 2: Fe(III)-respiration, Equation 3: sulfate-respiration, Equation 4: methanogenesis)**.

Equation 1	2 CO_2_	+ 8 Fe^2+^		+ 14 H_2_O	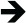	2 CH_2_O		+ 8 FeOOH		+ 16 H^+^
Equation 2	2 CH_2_O	+ 8 FeOOH		+ 8 H^+^	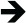	2 CO_2_		+ 8 Fe^2+^		+ 14 H_2_O
Equation 3	2 CH_2_O		+ SO^2−^_4_	+ 2 H^+^	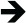	2 CO_2_			+ H_2_S	+ 2 H_2_O
Equation 4	2 CH_2_O				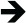	CO_2_	+ CH_4_			
Equation 1 + 3^a^		8 Fe^2+^	+ SO^2−^_4_	+ 12 H_2_O	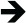			8 FeOOH	+ H_2_S	+ 14 H^+^
Equation 1 + 4	CO_2_	+ 8 Fe^2+^		+ 14 H_2_O	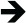		CH_4_	+ 8 FeOOH		+ 16 H^+^

*Assuming complete degradation of the biomass produced in Equation 1, only degradation through sulfate-reduction (Equation 1 + 3) and methanogenesis (Equation 1 + 4) will result in a net accumulation of Fe(III)-oxide minerals in the sediment. The fraction of degraded organic matter released as methane into the atmosphere represents a loss of reducing power and will consequently determine the amount of Fe(III) accumulating in the sediment*.

a *Sulfide will react chemically with excess Fe(III)oxides to form elemental sulfur and eventually pyrite, resulting in partially sulfidized Fe(III)-oxide deposits. Additional input of organic matter from anoxygenic photosulfidotrophs and oxygenic cyanobacteria would generally stimulate anaerobic degradation processes (Equations 2, 3, 4) and increase the degree of sulfidization of the sedimentary Fe-pool*.

While recent experimental work is not in favor of a significant contribution of photochemical processes to BIF formation (Konhauser et al., 2007), microbial Fe(II) oxidation remains an appealing possibility (Konhauser et al., 2002; Posth et al., 2008), especially for periods prior to the evolution of oxygenic photosynthesis. Although the evolution of photosynthesis is complex with horizontal gene transfer playing an important role, it is now accepted that anoxygenic phototrophic bacteria evolved before oxygen-producing cyanobacteria (Xiong et al., 2000; Raymond et al., 2003). The isolation of phototrophic Fe(II)-oxidizing bacteria (Widdel et al., 1993; Heising et al., 1999) has allowed the study of the influence of light intensity on iron oxidation and the role of temperature on the alternating precipitation of iron oxides and silica (Posth et al., 2008). Yet experimental field work aimed at elucidating the role of phototrophic Fe(II)-oxidation under natural environmental conditions as they may have existed in the chemocline of an Archean ferruginous ocean is still strongly needed (Johnston et al., 2009; Severmann and Anbar, 2009). Ferruginous water columns are rare, largely unexplored ecosystems of which only freshwater representatives exist today because of the high sulfate concentrations in the modern ocean. Recently, the presence of green anoxygenic phototrophic bacteria in the water column of a late Archean Ocean analog (Lake Matano, Indonesia) has been reported, and it has been suggested that they may be involved in Fe(II)-oxidation (Crowe et al., 2008). However, the respective activity could not be shown unambiguously and direct evidence for photoferrotrophic activity in a recent water column and quantitative data on their contribution to Fe(II)-oxidation are lacking to date (Crowe et al., 2014).

To address this, we investigated microbial iron cycling in the water column of Lake La Cruz (Rodrigo et al., 2001) in the Central Iberian Ranges (Spain), a permanently stratified lake ecosystem with a chemocline in the euphotic zone and a water column chemistry matching the putative late Archean conditions (Table 2). Using combined microbiological and biogeochemical approaches we assessed whether anoxygenic phototrophic Fe(II)-oxidizing microorganisms (photoferrotrophs) indeed thrive in the chemocline of an Archean ocean analog and contribute to the production of Fe(III) in an anoxic environment.

**Table 2:** **Comparison of water chemistry, iron fluxes toward the oxic/anoxic interface, and Fe(II)-oxidation rates of different potential modern Archean Ocean analogs**.

	**Lake La Cruz**		**Lake Matano (Crowe et al., 2008)**	**Lake Pavin (Bura-Nakic et al., 2009)**	**Archean Ocean**
	**Mixed layer**	**Anoxic layer**	**Anoxic layer**	**Anoxic layer**	**Anoxic layer**
Fe(II) (μM)	–	230	140	1000	40–120 (Crowe et al., 2008)
SO^2−^_4_ (μM)	<35	<25	<0.1	<5.0	≈80 (Jamieson et al., 2013)
O_2_ (μM)	238	0	0	0	<0.03 (Crowe et al., 2008)
PO^3−^_4_ (μM)	<0.26	<1.6	9	–	0.03–0.29 (Crowe et al., 2008)
pH	8.60	7.00	7.00	6.08	>6.5 (Crowe et al., 2008)
T° (°C)	16	6	25–28	4	≈36 (Crowe et al., 2008)
Fe(II) flux (μmol cm^2^ d^−1^)	0.031–0.244		0.034–0.27	–	12.3^a^
Estimated *In-situ* Fe(II) oxidation rate (μmol l^−1^ d^−1^)	0.174–1.396^b^		0.034–0.27	–	14 (Kappler et al., 2005)
Fe(II) oxidation rate (“^14^C”) (μmol l^−1^ d^−1^)	2.56^c^				
Fe(II) oxidation rate (“*ex-situ*”) (μmol l^−1^ d^−1^)	63.6^d^		–	–	
Number of cells (cell ml^−1^)	0.5 × 10^5^, 7.0 × 10^5^^e^		0.3–16 × 10^9^	–	10^6^ (Kappler et al., 2005)

a *Calculation based on the surface of Hamersley Basin (10^11^ m^2^) and a maximum Fe(III) precipitation rate of 4.5 × 10^12^ mol Fe(III) y^−1^ required to form the Hamersley Basin BIFs (Kappler et al., 2005)*.

b *Calculation see Table 3*.

c *Calculation based on the amount of ^14^C-bicarbonate fixed in “Fe(II) + DCMU” treatments (1.36 μg C l^−1^ h^−1^, **Figure 3**) minus the “No addition + DCMU” treatment (0.72 μg C l^−1^ h^−1^) and assuming a ratio of 4 Fe(II) oxidized per CO_2_ assimilated as shown in equation 1 (Table 1). Calculated for 12 h illumination per day*.

d *Calculation based on the average iron-oxidation rate of 2.65 μmol l^−1^ h^−1^ determined in the ex-situ light incubation with 12 h of illumination per day (**Figure 5**) and assuming that all Fe(II) was oxidized through photoferrotrophy*.

e *Maxima of GSB and PSB microscopic cell counts during summer stratification (Figure 1B)*.

## Materials and methods

### Physico-chemical profiling

Water column profiles of temperature, conductivity, pH, redox potential, dissolved oxygen, and chlorophyll-*a* were recorded using a Sea-Bird CTD multiprofiler. Light intensity was measured as photosynthetically active radiation (PAR) scalar irradiance with a flat Li-Cor Quantum Sensor (Li 192 SA), which was vertically mounted on a lowering frame and connected to a Li-Cor data logger (L-1000). Water samples were collected using a battery-driven peristaltic pump from a boat fixed in the center of the lake. Samples for Fe analysis were preserved with HCl (0.5 M final concentration), and samples for sulfide analysis with zinc acetate 5% (w/v). Concentrations of iron and sulfide were determined by the ferrozine (Viollier et al., 2000) and Cline (1969) assays, respectively. Solid phase iron and sulfur species (AVS and CRS) have been determined as described elsewhere (Haller et al., 2011). Major anions were determined by ion-chromatography on a DIONEX DX-120 system using an IonPac AS14A anion exchange column, Na_2_CO_3_/NaHCO_3_ (8 mM/1 mM) as eluent, an Anion Self-Regenerating Suppressor (ASRS 300, 4 mm) module, and a conductivity detector (Haller et al., 2011).

### Water samples

During summer stratification (13–17 October 08) samples for both *in-situ* and *ex-situ* incubation experiments were collected at 11.8 m depth, the euphotic and anoxic part of the chemocline (CAP), where both purple and green anoxygenic sulfur bacteria were present. At that time the CAP extended from 11.5 to 12.5 m depth. At 11.8 m depth, 1.8 μM O_2_, 1.4 μM Fe(II), 2.5 μM Fe(III), no H_2_S and a pH of 8.3 were measured. In winter (7–12 February 08) the CAP was between 14.5 and 15.5 m depth, whereby samples for the incubation experiments were collected from 15 m. The water at this depth was characterized by the absence of oxygen, 5 μM Fe(II), 1.8 μM Fe(III), 1.3 μM H_2_S and a pH of 8.2.

### Flux calculations

The vertical flux (*F_z_*) of Fe(II) toward the oxic/anoxic interface was calculated according to *F_z_* = −*K_z_* ^*^(Δ*C*/Δ*z*), assuming a linear concentration gradient between 15 and 18 m. As values for vertical eddy diffusivities (K_*z*_) we used 5.0 × 10^−4^ and 4.0 × 10^−3^ cm^−2^ s^−1^, which represent the reported minimum values for the iron-rich 95 m deep meromictic crater Lake Pavin, and the euxinic 20 m deep meromictic Lake Cadagno, both resembling Lake La Cruz (Bura-Nakic et al., 2009; Dahl et al., 2010). An upward flux of 0.03-0.24 μmol cm^2^ d^−1^ of Fe(II) toward the oxic/anoxic interface was calculated. In Table 2, La Cruz water column data are compared with conditions proposed for an Archean Ocean, and with potential modern analogs such as Lake Matano and Lake Pavin. Table 3 contains the parameters used for the Fe(II) flux calculations.

**Table 3:** **Calculation of ferrous iron-oxidation rates and fluxes toward the oxic/anoxic interface in Lake La Cruz under summer stratification conditions (October 2008)**.

Fe(II) gradient μmol cm^−4^	K_*Z*_ cm^2^ s^−1^	Fe(II) flux μmol cm^−2^ d^−1^	Fe(II)-oxidation^a^ μmol l^−1^ d^−1^
7.07 × 10^−04^	0.0005	0.031	0.174
	0.0040	0.244	1.396

a *Fe(II)-oxidation rate calculated for a 1.75 m thick water column layer and a 24 h day. The upper boundary of this zone was defined by the complete disappearance of Fe(II) at 11.25 m and the lower boundary by the limit of the secondary Fe(III) peak at 13 m depth*.

### *In-situ* bicarbonate-uptake experiments

Modified *in-situ* ^14^C-bicarbonate incubations were used to assess the influence of different substrates potentially involved in microbial iron and sulfur cycling. For this, water was sampled during summer stratification (CAP: 11.8 m). Incubations were performed in 10 ml N_2_-preflushed Vacutainer tubes (Becton Dickinson) with the following additions from anoxic stock solutions (final concentrations): (i) untreated control, (ii) FeCl_2_(100 μM), (iii) NaNO_3_ (50 μM), (iv) sulfide (100 μM). The pH change in the incubation tube was negligible when the different amendments were added (e.g., drop from pH 8.3 to pH 8.2 upon FeCl_2_ addition). All of the treatments were done in duplicates, with and without DCMU, as well as under light and dark conditions. For the incubations with DCMU, the water sample was treated with the inhibitor 30 min prior the start of the experiment assuring its effect on photosystem II (PSII). The tubes were spiked with 5 μCi of anoxic ^14^C-bicarbonate (DHI, Hørsholm, Denmark) and immediately incubated for 4 h at 10 m depth (2% PAR, 12.6 °C), corresponding to the upper boundary of the Chl *a* peak, in order to avoid shading of the different phototrophic guilds. After incubation, samples were killed with formalin (2% final concentration) and filtered (GF/F Whatman). Filters were rinsed twice with 0.05 M HCl and finally with milliQ water. After a drying period of 36 h, filters were counted in 10 ml of Ultima Gold aqueous scintillation liquid on a Wallac 1409 liquid scintillation analyzer. Rates were calculated according to: Uptake rate = ^14^C-fixed × [ΣCO_2_] × 1.06/Σ^14^CO_2_ × *t*, where ^14^C-fixed is the radioactivity counts per filter minus control, [ΣCO_2_] is the total DIC concentration in the water at the sampling depth, 1.06 is the correction factor for isotopic fractionation between ^12^C and ^14^C, Σ^14^CO_2_ is the total DIC radioactivity per vial, and *t* is the incubation time. The net photosynthetic carbon uptake was obtained by subtracting the dark incubation values from the carbon uptake in the light. Chemoautotrophy can be higher in samples incubated in the dark than in the ones incubated in light, due to competition for nutrients with phototrophs in illuminated bottles. Therefore, we consider the presented phototrophic carbon uptake value as the minimum for photoautotrophy. Uptake rates were used to build two generalized linear models (-C- uptake ~Fe + NO^−^_3_ + H_2_S)(Maccullagh and Nelder, 1989) with a binomial distribution, once with DCMU additions and once without. The influence of the treatments on carbon uptake was tested using ANOVA. All analyses were performed using the statistical software “R” (R Development Core Team, 2009).

### *Ex-situ* Fe(II) oxidation experiments

N_2_-flushed 500 ml bottles were filled with water from 11.8 m depth and closed anoxically with thick butyl rubber stoppers. The samples were directly transported to the laboratory at the University of Valencia under dark and cool conditions. After addition of FeCl_2_ to a final concentration of ≈500 μM (shifting the pH from 8.3 to 7.8), bottles were incubated with and without DCMU at 14°C for 120 h. Half of the bottles were incubated under a 12 h light-dark regime with a direct light intensity of 61 μE m^−2^ s^−1^; the rest were kept under continuous dark conditions. For the bottles with DCMU, 10 ml of an anoxic, saturated aqueous solution of DCMU were injected 1 h before the FeCl_2_ addition to inhibit PSII of oxygenic phototrophs. The evolution of ferric and ferrous iron was followed by the ferrozine assay (Viollier et al., 2000). A killed control was incubated under continuous light to maximize any potential photo-oxidative effects on iron speciation. Photocatalytic Fe(II)-oxidation, however, was not observed. The appearance of Fe(III) in the dark incubation (**Figure 5B**) toward the end of the experiment may be explained by O_2_ contamination upon sampling. The chosen Fe(II) concentration was within the range predicted for an Archean Ocean (0.05–0.54 mM) (Holland, 1973; Croal et al., 2009) yet higher than the *in situ* concentration. Hence, the determined oxidation rates represent potential rates, valid for replete Fe(II) and light conditions, and are a measure for the population size of photoferrotrophs.

### Enrichment and molecular identification of photoferrotrophs

Enrichments were established with CAP water collected at 15 m in February 2008 (see water sampling). A sample of 200 ml was anoxically transferred to a sterile, N_2_-flushed serum bottle and amended with 200 μM FeCl_2_ and DCMU. The bottle was incubated at room temperature under a 12 h light-dark regime for 1 month. A sub-sample was afterwards transferred into bicarbonate-buffered (pH 6.8) freshwater mineral medium (Heising et al., 1999) that contained 10 mM FeCO_3_, trace elements, and vitamins including vitamin B_12_. After an initial serial dilution to extinction, the tubes were exposed to 61 μE m^−2^ s^−1^ direct light (12 h L/D). For the phylogenetic characterization of the photoferrotrophic culture a clone library targeting the 16S rRNA gene was established after the 5th enrichment transfer. Thirty-one clones were sequenced after a prior restriction fragment analysis using *Hae*III and *Taq*I. The nearly complete 16S rRNA sequences were aligned in ClustalW and manually refined (1344 positions). The phylogenetic tree is based on the results of a maximum likelihood analysis of sequences from the *Chlorobiaceae* family, including *C. clathratiforme* the dominant green sulfur bacterium in this lake (Rodrigo et al., 2001; Romero-Viana et al., 2010). The phylogenetic tree was created by using GTR (General Time Reversible) model +Γ + invariant and 4 substitution rate categories (1000 bootstrap) using Treefinder (Jobb et al., 2004). Sequences were submitted to the NCBI sequence database under accession numbers FN4994988–FN994991.

### Fe(II)-oxidation by the photoferrotrophic enrichment culture

Hungate tubes (10 ml) containing the same medium as described above were inoculated with the enrichment culture, that had been sub-cultivated for 9 months (corresponding to 5 enrichment transfers) before using it for the experiment. The inoculated tubes were incubated in triplicates at 20°C under continuous dark and continuous light conditions (70 μE m^−2^ s^−1^). The photometric ferrozine assay (Viollier et al., 2000) was used to follow the evolution of Fe(II)/Fe(III) in the tubes. Average Fe(II) oxidation rates were calculated from the Fe(II) concentration changes between day 7, corresponding to the end of Fe(III) reduction phase, and day 60.

### Quantification of phototrophic organisms

Algae were counted by the Utermöhl sedimentation method and picocyanobacteria by epifluorescence microscopy. The biomass of oxygenic phototrophs was calculated from algal and picocyanobacterial cell counts and the corresponding biovolumes (data not shown). In Lake La Cruz, PSB are mostly *Lamprocystis purpurea*, and GSB are mostly *Chlorobium clathratiforme*. Their cell numbers were determined by filtering water samples through 0.2 μm pore-size membrane filters, followed by erythrosine staining, and counting on a Zeiss III phase-contrast microscope. Additionally, the biomass of PSB and GSB was calculated from replicated counts after determination of cell biovolumes (data not shown). Chlorophylls and bacteriochlorophylls were determined by RP-HPLC. Details of all procedures have been published elsewhere (e.g., Miracle et al., 2000).

### Estimation of the population density of photoferrotrophs and their proportion within the GSB

The number of photoferrotrophs in the *ex situ* incubations was estimated by dividing the measured Fe(II)-oxidation rate of 2.65 μmol l^−1^ h^−1^ (Table 2) by the cell specific Fe(II)-oxidation rate. No published data are available for *Chlorobium ferrooxidans*. We used therefore a rather low value from *Rhodobacter ferrooxidans* SW2 (32 pmol Fe(II) h^−1^ cell^−1^) (Hegler et al., 2008) in order not to underestimate the population size of photoferrotrophs. Since there were about 0.03 × 10^5^ GSB cells ml^−1^ in the water that has been used for the incubation experiments it follows that about 3% of the GSB present at that depth act indeed as photoferrotrophs.

## Results and discussion

Most meromictic lakes and other permanently stratified water bodies are euxinic, i.e., anoxic and sulfidic below the chemocline (Lyons et al., 2009). Lake La Cruz is a rare exception as its anoxic bottom water contains little sulfide but is rich in dissolved Fe(II) (Figure 1A). Sulfate concentrations are low (<35 μM; Figure 1A) due to the low sulfur content of the surrounding dolomite rocks and marlstones (Rodrigo et al., 2001). Dissolved sulfide was detected just below the chemocline, originating from decaying organic matter (Romero-Viana et al., 2010) and dissimilatory sulfate reduction as indicated by decreasing sulfate concentrations with depth and 16S rRNA gene sequences of sulfate -reducing bacteria (*Desulfomonile* sp.) in a clone library from the anoxic part of the chemocline (Walter, 2011). Sulfide concentrations were very low, even though they were determined with the photometric Cline assay, which overestimates free sulfide concentrations as it detects also colloidal and amorphous forms of FeS (Bura-Nakic et al., 2009). Most of the iron in the anoxic water column was thus present as dissolved Fe(II). Fe(II) reached the chemocline at a rate of 0.031–0.244 μmol cm^2^ d^−1^ (Tables 1, 2), where it was oxidized as indicated by two separate Fe(III) maxima (Figure 1A).

**Figure 1:**
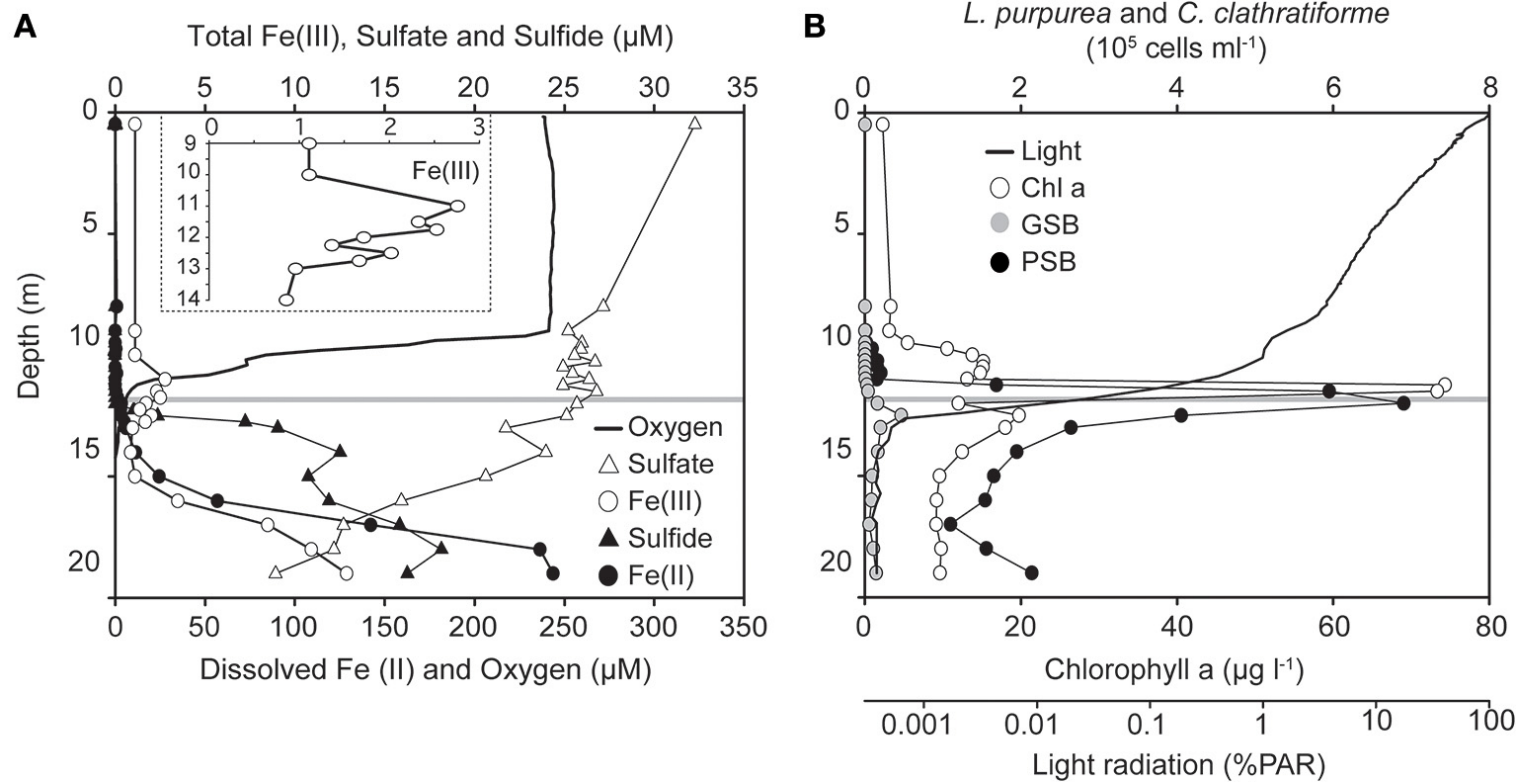
**Summer stratification water column data of Lake La Cruz. (A)** Chemical stratification at the time when incubation experiments were performed (13/Oct/2008). The insert depicts a magnified Fe(III) concentration profile around the chemocline. **(B)** Vertical distribution of oxygenic (chlorophyll *a* concentration, open circles) and anoxygenic phototrophs. Black symbols stand for microscopic cell counts of the dominant purple sulfur bacterium (PSB) *Lamprocystis purpurea)* and gray symbols for the dominant green sulfur bacterium (GSB) *Chlorobium clathratiforme*. The horizontal line at 11.8 m indicates the sampling depth for the *in situ* radiocarbon-incubations and *ex situ* Fe(II)-oxidation experiments.

**Figure 2:**
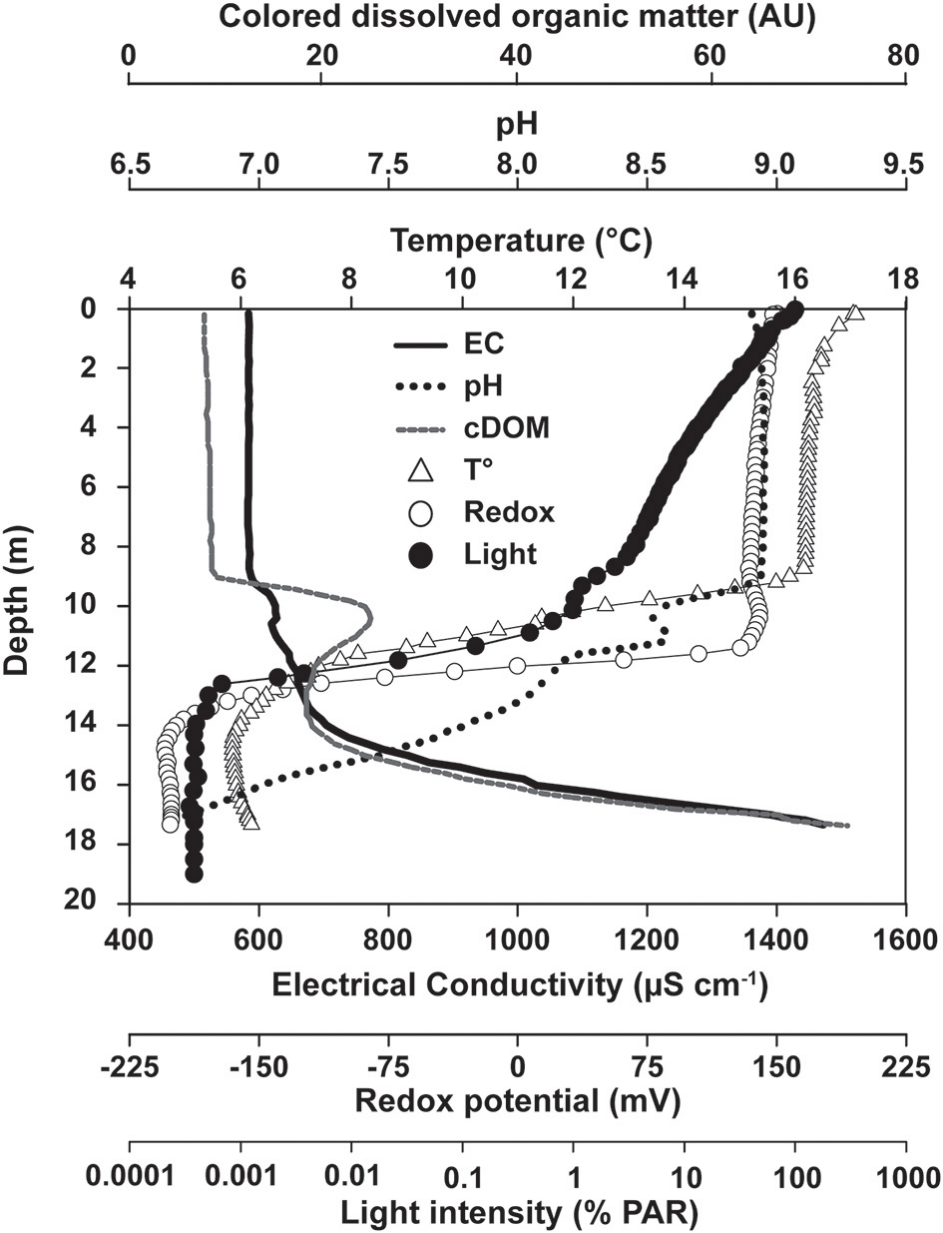
**Depth profiles of physico-chemical conditions in the water column of Lake La Cruz**. Profiles were recorded during summer stratification conditions (13/Oct/2008).

An upper broad peak of 2.8 μM Fe(III) was located between 10 and 11.75 m, where picocyanobacteria were most abundant and Fe(III) was presumably formed by direct chemical reaction with O_2_ or by microaerophilic chemotrophs (Lehours et al., 2007). As nitrate was also present at that depth (2.0 μM), chemotrophic nitrate-dependent iron-oxidation cannot be excluded, but appeared to be of minor importance (Walter, 2011), possibly due to the limited supply of this oxidant as well as competition for nitrate with denitrifying bacteria and nitrate-assimilating phototrophs. A second peak of Fe(III) (~2.0 μM, 12–13 m) was typically observed in the anoxic part of the chemocline and coincided with the biomass maxima of the anoxygenic phototrophs *Chlorobium clathratiforme* and *Lamprocystis purpurea* (Figures 1B, **8**). Prevailing light intensities of 0.02–0.002% PAR (Figures 1B, 2) and a continuous supply of Fe(II) from the hypolimnion constitute suitable conditions for the development of photoferrotrophs.

**Figure 3:**
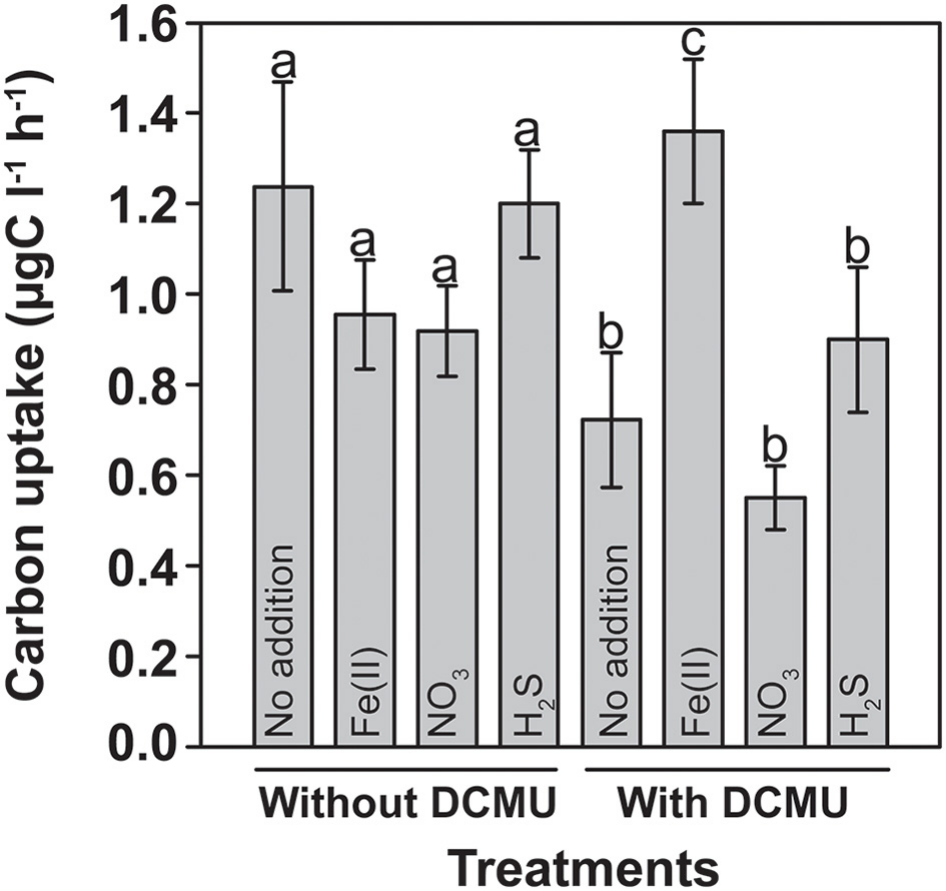
**Light-dependent inorganic carbon uptake in presence of potential substrates for lithoautotrophic iron- and sulfur-transforming microorganisms**. *In-situ* net photosynthetic carbon fixation experiments showing a statistically significant stimulation upon Fe(II) addition in presence of DCMU (-*c*-, *p* < 0.05) that inhibits oxygen production by photosynthesis. The net photosynthetic carbon uptake was obtained by subtracting the dark incubation values from the carbon uptake in the light. Letters represent the statistical groups using generalized linear models. Average values and standard deviations are presented (13/Oct/2008).

Photoferrotrophy being an autotrophic metabolism, *in-situ* ^14^C-incubation experiments were conducted to detect any Fe(II)-dependent stimulation of carbon uptake in the light. Incubations were performed with water samples from the anoxic part of the chemocline at 11.8 m, where sulfide and Fe(II) concentrations were minimal. The incubations were amended with various electron donors and acceptors, which may fuel autotrophic metabolism, including Fe(II), sulfide, and nitrate. Since both oxygenic and anoxygenic phototrophs were present at this depth (Figures 1B, 8), DCMU (3-{3,4-dichlorophenyl}-1,1-dimethylurea) was added to parallel incubations in order to suppress oxygen production by PSII. In the absence of DCMU, none of the additions had a statistically significant influence on carbon uptake (Figure 3). The inorganic carbon-uptake rates were generally 35–40% lower in the presence of DCMU, with the only exception of the Fe(II) treatment where a significant increase of 40% was observed (*P* < 0.05) and where the highest fixation rates were determined among all assayed conditions (Figure 3).

**Figure 4:**
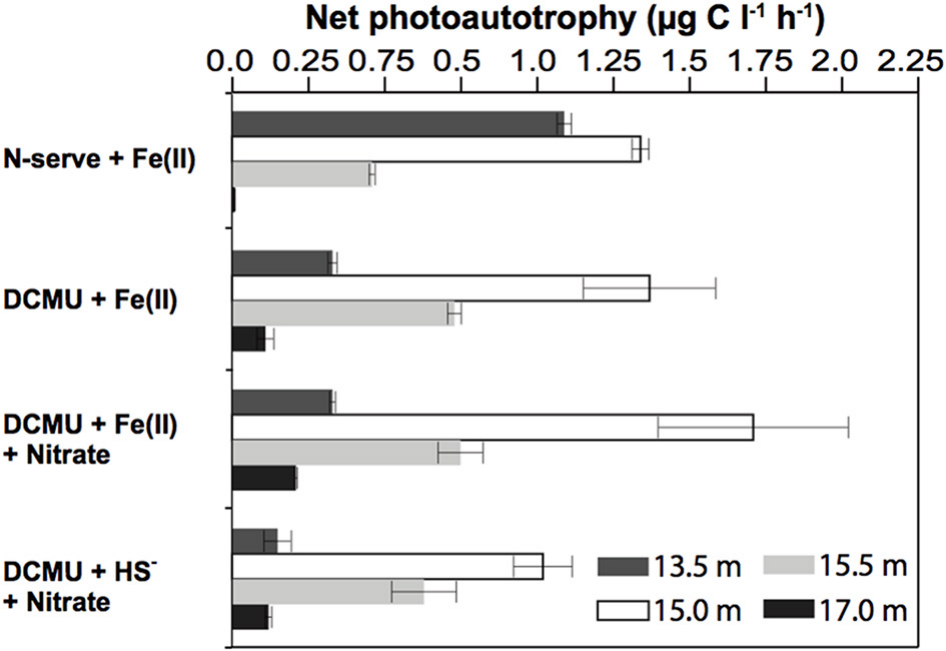
***In situ* light dependent inorganic carbon uptake in presence of potential substrates for iron- and sulfur-transforming microorganisms**. Results show highest stimulation of bicarbonate uptake upon Fe(II) addition at 15 m and 15.5 m, the depths where anoxygenic phototrophs were most abundant. Net photosynthetic carbon uptake was obtained by subtracting the dark incubation values from the carbon uptake in the light. N-serve and DCMU were added to inhibit nitrification and PS(II) of oxygenic phototrophs, respectively. Means of duplicate experiments are given, with bar ends indicating highest and lowest values. Winter stratification conditions (11/Feb/2008).

This result suggests that there was Fe(II)/light-dependent inorganic carbon uptake in the anoxic part of the chemocline, and that oxygenic photosynthesis needed to be inhibited to detect photoferrotrophic autotrophy. It has been proposed that ancestral cyanobacteria could photosynthesize with PS I alone and probably used H_2_, H_2_S, or Fe(II) to reduce CO_2_ to organic matter (Pierson, 1994). Also some modern cyanobacteria may switch in response to the environmental conditions from oxygenic to anoxygenic photosynthesis with H_2_S (Cohen et al., 1986). A contribution of cyanobacteria to the observed stimulation in presence of DCMU cannot be entirely excluded, although such metabolic versatility was never found in picocyanobacteria (Pierson et al., 1999) such as those being abundant in Lake La Cruz. Similar incubations done during winter, when the different phototrophic populations were less compact and better separated in the water column, revealed that fuelling of ^14^C-uptake by Fe(II) was strongest in water layers where anoxygenic phototrophs were present (15 and 15.5 m). Conversely, only a weak stimulation was observed in samples from the upper cyanobacterial layer (13.5 m) or the aphotic monimolimnion (17 m), respectively (Figure 4).

**Figure 5:**
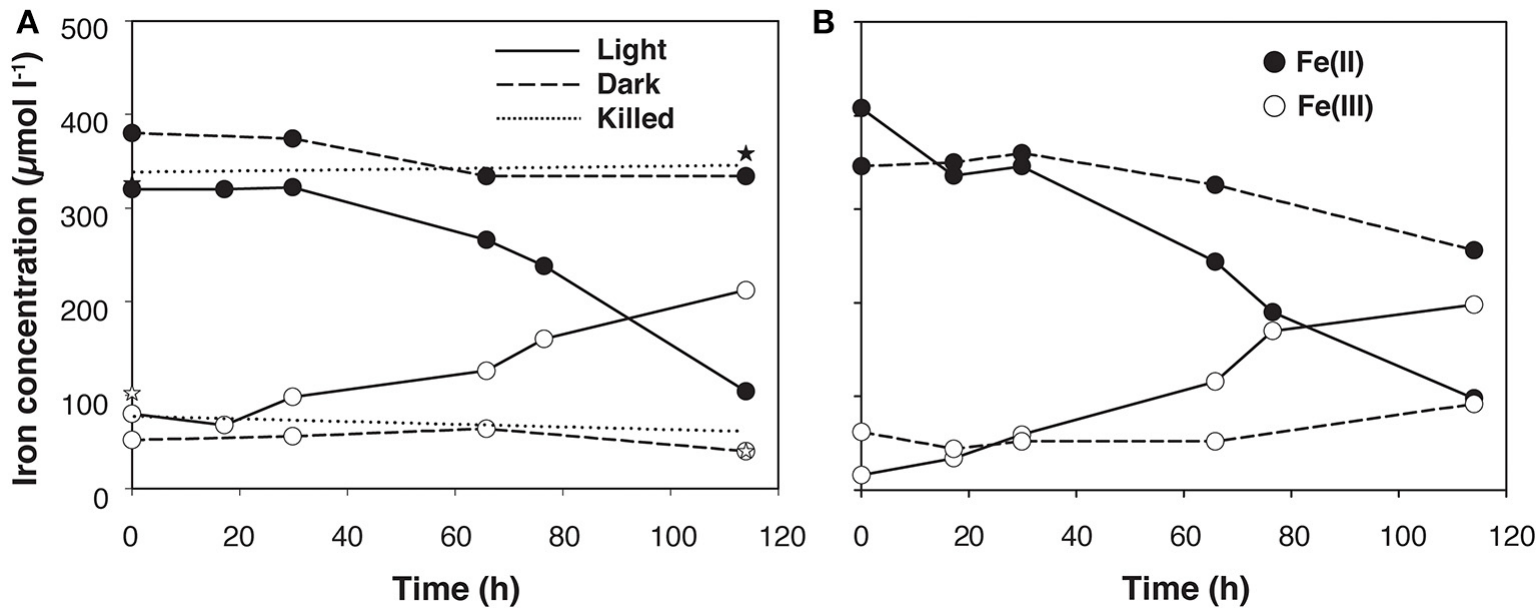
**Light-dependent iron-oxidation by the natural microbiota from the anoxic part of the Lake La Cruz chemocline at 11.8 m depth**. Anoxic laboratory (*ex situ*) incubation experiment (11/Feb/2008) **(A)** without DCMU addition, where oxygenic photosynthesis is active and abiotic Fe(II) oxidation with O_2_ may occur; and **(B)** Fe(II) evolution under anoxic conditions in absence of oxygenic photosynthesis (with DCMU addition). For both experimental settings, light conditions involved consecutive periods of 12 h illumination at 61 μE m^−2^ s^−1^ and 12 h darkness. Solid symbols stand for Fe(II) and open symbols for Fe(III) concentrations. The dotted lines represent means of killed controls (stars; *n* = 3).

**Figure 6:**
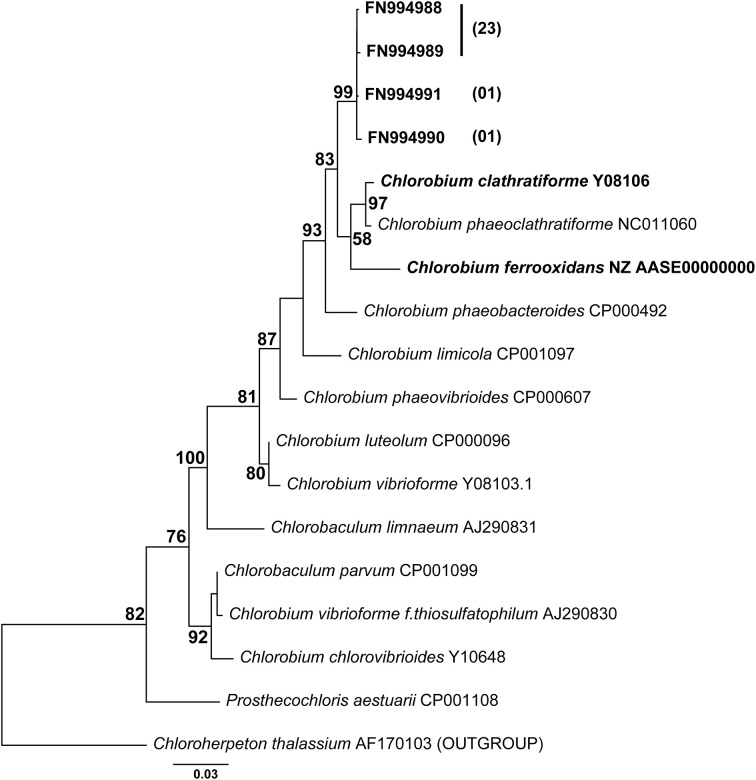
**Phylogeny of the photoferrotrophic enrichment culture based on nearly complete 16S rRNA gene sequences and maximum likelihood analysis**. Among the retrieved sequences 80% formed a well-defined cluster within the *Chlorobia* (25/31 clones), phototrophic green sulfur bacteria; the rest of the sequences were most closely related to uncultivated *Acidobacteria* (5/31 clones). Numbers in brackets signify the number of clones possessing the same sequence. Bootstrap values >50% for 1000 replications are shown.

**Figure 7:**
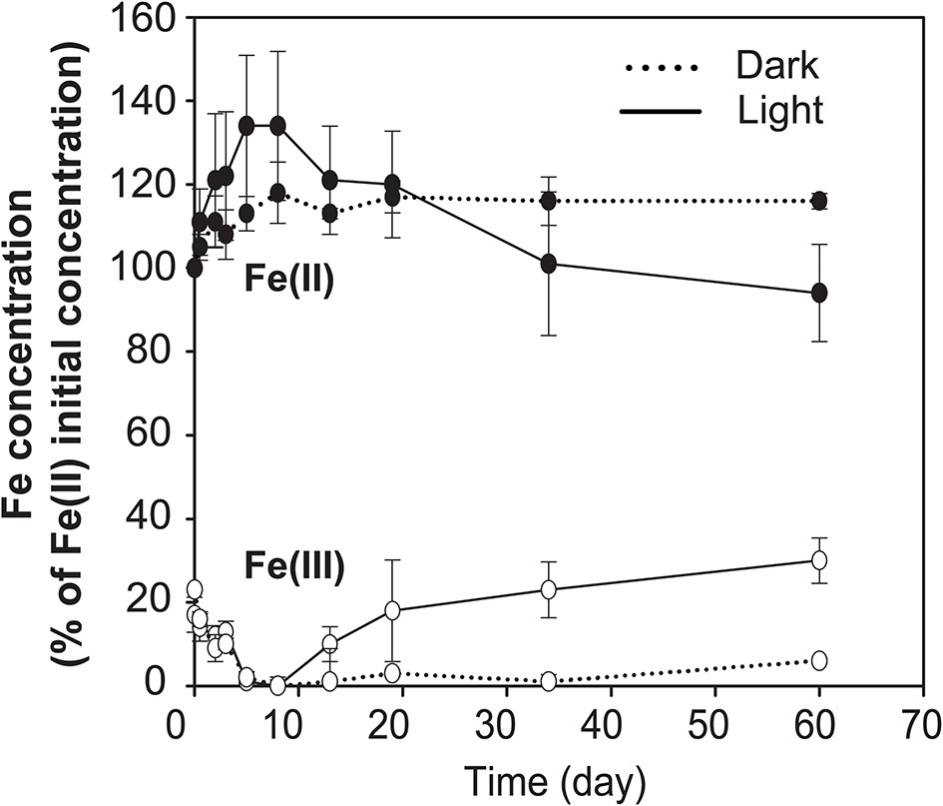
**Net Fe(II)-oxidation by the photoferrotrophic enrichment culture**. Results display the dependence of Fe(II)-oxidation on light. Furthermore, Fe(III) added with the inoculum at the beginning of the experiment was reduced during the first 8 h, presumably mediated by *Acidobacteria*, the *second* most abundant bacterial group in the enrichment culture. Data are average values of three independent experiments with error bars representing standard deviations (*n* = 3).

No data on rates of anoxygenic phototrophic Fe(II)-oxidation in an Archean Ocean analog existed so far. Anaerobic light dependent oxidation of iron was quantified using *ex-situ* incubations with Fe(II)-enriched water from the same depth as used for the ^14^C-incubations (Figure 5). The oxidation of Fe(II) to Fe(III) by the natural chemocline microbiota was light dependent and occurred at a potential rate of 2.7 μmol l^−1^ h^−1^. The rate was similar for incubations with and without DCMU, alluding to the fact that oxygenic phototrophs (e.g., picocyanobacteria) from this depth were photosynthetically not very active, as also demonstrated by parallel studies investigating the annual cycle of inorganic carbon assimilation (Picazo, personal communication). Moreover, this estimate shows that the population size of photoferrotrophs in the chemocline was high enough to reach, under non limiting light conditions, Fe(II) oxidation rates believed to be required for BIFs formation (Kappler et al., 2005) (Table 2). Alternative Fe(II) oxidation rate estimates based on equation (1) and the amount ^14^CO_2_ incorporated *in situ*, under Fe(II)-enriched conditions, amounted only to 0.2 μmol Fe(II) l^−1^ h^−1^ (Table 2). This was likely due to the lower light intensities available in the lake. Alternatively, it may also indicate that photoferrotrophs in Lake La Cruz are not obligate autotrophic organisms and that they could assimilate additional organic compounds for biomass formation (Heising et al., 1999). Our attempts to cultivate phototrophic Fe(II)-oxidizing organisms from chemocline water samples resulted in a co-culture consisting of *Chlorobium* sp. (Figure 6, 80%) and as yet uncultivated *Acidobacteria* (20%). The *Chlorobium* strain was closely related to *Chlorobium ferrooxidans*, the only green photoferrotrophic culture known so far, and to *Chlorobium clathratiforme*, the dominant green phototrophic sulfur bacterium in La Cruz (Rodrigo et al., 2001; Romero-Viana et al., 2010). The enrichment culture contained both photoferrotrophic and Fe(III)-reducing bacteria (Figure 7) suggesting that Fe(II)-oxidizing and reducing processes in the chemocline of Lake Cruz are tightly coupled. The observed Fe(II) oxidation rate of 2.6 μmol l^−1^ h^−1^ represents thus a net rate, depending on the relative kinetics of the processes, and falls into the lower range of what has been determined in other cultures of Fe(II)-oxidizing phototrophs (Hegler et al., 2008).

**Figure 8:**
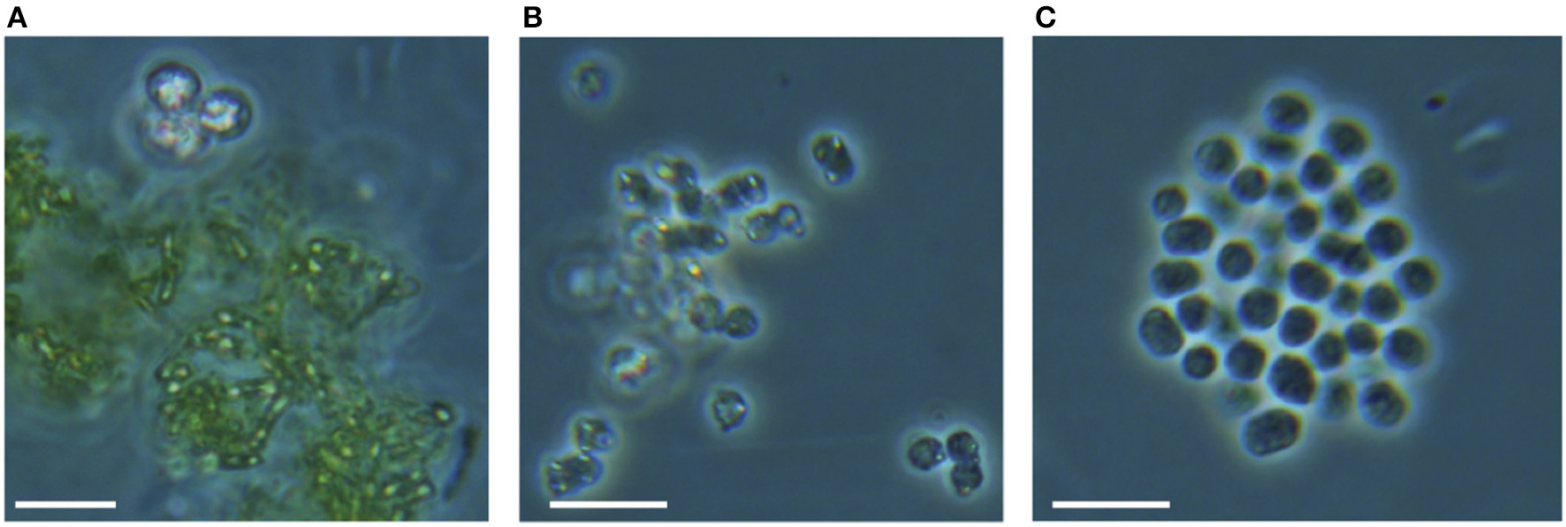
**Photomicrographs of (A) green and (B,C) purple anoxygenic phototrophs from the chemocline of Lake La Cruz**. They show yellowish sulfur globules inside of PSB cells or deposited around GSB cells **(A,B)**. PSB cells without apparent internal S^0^ deposits are also frequently observed **(C)**. Scale bars represent 10 μm.

Despite complete oxidation of Fe(II) at the chemocline and hence a continuous flux of sedimenting Fe(III) to the bottom of the lake (Figure 1A), there was no accumulation of iron oxide minerals in the sediments. Only 3 μmol Fe(III) g^−1^ w/w was detected at the sediment surface (data not shown). Below 0.5 cm depth, HCl extractable iron was reduced and bound to sulfur as suggested by combined iron and sulfur measurements (data not shown). Iron sulfide is produced continuously just below the chemocline and settles down the water column (Ma et al., 2006), however, in contrast to Fe(III)-oxides, which can be reduced back to Fe^2+^, FeS is stable and will accumulate in the sediment. Iron sulfide minerals are also produced at the sediment surface from Fe(III)-oxides reacting with sulfide liberated through organic matter degradation and dissimilatory sulfate reduction. An accumulation of Fe(III) in the sediment would only be possible with a strongly reduced primary productivity and significant degradation of organic matter by methanogenesis during sedimentation (Table 1). We propose therefore that periods of BIF formation under anoxic Archean conditions were associated with increased methane formation, which is consistent with the current notion of biogenic methane being an important component of the Archean atmosphere (Zerkle et al., 2012).

## Conclusion

Chemical profiles of iron with a recurrent secondary peak of Fe(III) in the anoxic, euphotic part of the chemocline, along with the increased inorganic carbon-uptake in presence of Fe(II), and the light-dependent Fe(II) oxidation, provide consistent evidences for photoferrotrophic activity in the La Cruz chemocline, and represent a proof of concept for their possible contribution to ancient BIF formation prior to the evolution of oxygenic photosynthesis. However, we note that photoferrotrophy under the prevailing environmental conditions in La Cruz is a slow process and that most of the Fe(II) at the chemocline is oxidized by molecular oxygen. Moreover, photoferrotrophs represent only a minor fraction of the anoxygenic phototrophic community with the majority apparently thriving by sulfur cycling, despite the very low sulfide and sulfate contents in the ferruginous water column of Lake La Cruz. This observation is also supported by a recent publication that showed that a cryptic sulfur cycle can occur in ferruginous conditions (Crowe et al., 2014). We hypothesize therefore that cryptic sulfur cycling, as recently shown for oxygen minimum zones in upwelling areas of the modern Ocean (Canfield et al., 2010), was also a feature of the late Archean Ocean where predicted sulfate concentrations were 3–10 times higher than in Lake La Cruz (Canfield, 2005; Jamieson et al., 2013).

### Conflict of interest statement

The authors declare that the research was conducted in the absence of any commercial or financial relationships that could be construed as a potential conflict of interest.

## References

[B1] Anbar A. D. Knoll A. H. (2002). Proterozoic ocean chemistry and evolution: a bioinorganic bridge? Science 297, 1137–1142. 10.1126/science.1069651 12183619

[B2] Braterman P. S. Cairns-Smith A. G. Sloper R. W. (1983). Photooxidation of hydrated Fe-2+ - significance for banded iron formations. Nature 303, 163–164 10.1038/303163a0

[B3] Bura-Nakic E. Viollier E. Jezequel D. Thiam A. Ciglenecki I. (2009). Reduced sulfur and iron species in anoxic water column of meromictic crater Lake Pavin (Massif Central, France). Chem. Geol. 266, 320–326 10.1016/j.chemgeo.2009.06.020

[B4] Canfield D. E. (2005). The early history of atmospheric oxygen: homage to Robert A. Garrels. Annu. Rev. Earth Planet. Sci. 33, 1–36 10.1146/annurev.earth.33.092203.122711

[B5] Canfield D. E. Stewart F. J. Thamdrup B. De Brabandere L. Dalsgaard T. Delong E. F.. (2010). A cryptic sulfur cycle in oxygen-minimum-zone waters off the chilean coast. Science 330, 1375–1378. 10.1126/science.1196889 21071631

[B6] Cline J. D. (1969). Spectrophotometric determination of hydrogen sulfide in natural waters. Limnol. Oceanogr. 14, 454–458 10.4319/lo.1969.14.3.0454

[B7] Cloud P. E. (1968). Atmospheric and hydrospheric evolution on primitive earth. Science 160, 729–736. 10.1126/science.160.3829.729 5646415

[B8] Cohen Y. Jørgensen B. B. Revsbech N. P. Poplawski R. (1986). Adaptation to hydrogen-sulfide of oxygenic and anoxygenic photosynthesis among cyanobacteria. Appl. Environ. Microbiol. 51, 398–407. 16346996 10.1128/aem.51.2.398-407.1986 PMC238881

[B8a] Croal L. R. Jiao Y. Kappler A. Newman D. K. (2009). Phototrophic Fe(II) oxidation in an atmosphere of H2: implications for Archean banded iron formations. Geobiology 7, 21–24. 10.1111/J.1472-4669.2008.00185.X 19200143 PMC2763526

[B9] Crowe S. A. Jones C. Katsev S. Magen C. O'Neill A. H. Sturm A.. (2008). Photoferrotrophs thrive in an Archean Ocean analogue. Proc. Natl. Acad. Sci. U.S.A. 105, 15938–15943. 10.1073/pnas.0805313105 18838679 PMC2572968

[B10] Crowe S. A. Maresca J. A. Jones C. Sturm A. Henny C. Fowle D. A.. (2014). Deep-water anoxygenic photosythesis in a ferruginous chemocline. Geobiology 12, 322–339. 10.1111/gbi.12089 24923179

[B11] Dahl T. W. Anbar A. D. Gordon G. W. Rosing M. T. Frei R. Canfield D. E. (2010). The behavior of molybdenum and its isotopes across the chemocline and in the sediments of sulfidic Lake Cadagno, Switzerland. Geochim. Cosmochim. Acta 74, 144–163 10.1016/j.gca.2009.09.018

[B12] Haller L. Tonolla M. Zopfi J. Peduzzi R. Wild W. Pote J. (2011). Composition of bacterial and archaeal communities in freshwater sediments with different contamination levels (Lake Geneva, Switzerland). Water Res. 45, 1213–1228. 10.1016/j.watres.2010.11.018 21145090

[B13] Hegler F. Posth N. R. Jiang J. Kappler A. (2008). Physiology of phototrophic iron(II)-oxidizing bacteria: implications for modern and ancient environments. FEMS Microbiol. Ecol. 66, 250–260. 10.1111/j.1574-6941.2008.00592.x 18811650

[B14] Heising S. Richter L. Ludwig W. Schink B. (1999). *Chlorobium ferrooxidans sp.* nov., a phototrophic green sulfur bacterium that oxidizes ferrous iron in coculture with a “*Geospirillum*” sp. strain. Arch. Microbiol. 172, 116–124. 10.1007/s002030050748 10415173

[B15] Holland H. D. (1973). Oceans - possible source of iron in iron-formations. Econ. Geol. 68, 1169–1172. 10.2113/gsecongeo.68.7.116911542097

[B15a] Jamieson J. W. Wing B. A. Farquhar J. Hannington M. D. (2013). Neoarchaean seawater sulphate concentrations from sulphur isotopes in massive sulphide ore. Nat. Geosci. 6, 61–64 10.1038/ngeo1647

[B16] Jobb G. Von Haeseler A. Strimmer K. (2004). TREEFINDER: a powerful graphical analysis environment for molecular phylogenetics. BMC Evol. Biol. 4:18. 10.1186/1471-2148-4-18 15222900 PMC459214

[B17] Johnston D. T. Wolfe-Simon F. Pearson A. Knoll A. H. (2009). Anoxygenic photosynthesis modulated Proterozoic oxygen and sustained Earth's middle age. Proc. Natl. Acad. Sci. U.S.A. 106, 16925–16929. 10.1073/pnas.0909248106 19805080 PMC2753640

[B18] Kappler A. Pasquero C. Konhauser K. O. Newman D. K. (2005). Deposition of banded iron formations by anoxygenic phototrophic Fe(II)-oxidizing bacteria. Geology 33, 865–868 10.1130/G21658.1

[B19] Konhauser K. O. Amskold L. Lalonde S. V. Posth N. R. Kappler A. Anbar A. (2007). Decoupling photochemical Fe(II) oxidation from shallow-water BIF deposition. Earth Planet. Sci. Lett. 258, 87–100 10.1016/j.epsl.2007.03.026

[B20] Konhauser K. O. Hamade T. Raiswell R. Morris R. C. Ferris F. G. Southam G.. (2002). Could bacteria have formed the Precambrian banded iron formations? Geology 30, 1079–1082. 10.1130/0091-7613(2002)0302.0.CO;224606418

[B21] Lehours A. C. Evans P. Bardot C. Joblin K. Gerard F. (2007). Phylogenetic diversity of archaea and bacteria in the anoxic zone of a meromictic lake (Lake Pavin, France). Appl. Environ. Microbiol. 73, 2016–2019. 10.1128/AEM.01490-06 17261512 PMC1828810

[B22] Lyons T. W. Anbar A. D. Severmann S. Scott C. Gill B. C. (2009). Tracking euxinia in the ancient ocean: a multiproxy perspective and proterozoic case study. Annu. Rev. Earth Planet. Sci. 37, 507–534 10.1146/annurev.earth.36.031207.124233

[B23] Ma S. F. Noble A. Butcher D. Trouwborst R. E. Luther G. W. (2006). Removal of H2S via an iron catalytic cycle and iron sulfide precipitation in the water column of dead end tributaries. Estuar. Coast. Shelf Sci. 70, 461–472 10.1016/j.ecss.2006.06.033

[B24] Maccullagh P. Nelder J. A. (1989). Generalized Linear Models. London: Chapman & Hall.

[B25] Miracle M. R. Camacho A. Julià R. Vicente E. (2000). Sinking processes and their effect on the sedimentary record in the meromictic Lake La Cruz (Spain). Verh. Int. Ver. Limnol 27, 1209–1213.

[B26] Pierson B. K. (1994). The emergence, diversification, and role of photosynthetic eubacteria, in Early Life on Earth, ed Bengtson S. (New York, NY: Columbia University Press), 161–180.

[B27] Pierson B. K. Parenteau M. N. Griffin B. M. (1999). Phototrophs in high-iron-concentration microbial mats: physiological ecology of phototrophs in an iron-depositing hot spring. Appl. Environ. Microbiol. 65, 5474–5483. 10584006 10.1128/aem.65.12.5474-5483.1999 PMC91746

[B28] Posth N. R. Hegler F. Konhauser K. O. Kappler A. (2008). Alternating Si and Fe deposition caused by temperature fluctuations in Precambrian oceans. Nat. Geosci. 1, 703–708 10.1038/ngeo306

[B29] R Development Core Team. (2009). R: A language and environment for statistical computing. Vienna: R Foundation for Statistical Computing.

[B30] Raymond J. Zhaxybayeva O. Gogarten J. P. Blankenship R. E. (2003). Evolution of photosynthetic prokaryotes: a maximum-likelihood mapping approach. Philos. Trans. R. Soc. Lond. B Biol. Sci. 358, 223–230. 10.1098/rstb.2002.1181 12594930 PMC1693105

[B31] Rodrigo M. A. Miracle M. R. Vicente E. (2001). The meromictic Lake La Cruz (Central Spain). Patterns of stratification. Aquat. Sci. 63, 406–416 10.1007/s00027-001-8041-x

[B32] Romero-Viana L. Keely B. J. Camacho A. Vicente E. Miracle M. R. (2010). Primary production in Lake La Cruz (Spain) over the last four centuries: reconstruction based on sedimentary signal of photosynthetic pigments. J. Paleolimnol. 43, 771–786 10.1007/s10933-009-9367-y

[B33] Severmann S. Anbar A. D. (2009). Reconstructing paleoredox conditions through a multitracer approach: the key to the past is the present. Elements 5, 359–364 10.2113/gselements.5.6.359

[B34] Viollier E. Inglett P. W. Hunter K. Roychoudhury A. N. Van Cappellen P. (2000). The ferrozine method revisited: Fe(II)/Fe(III) determination in natural waters. Appl. Geochem. 15, 785–790 10.1016/S0883-2927(99)00097-9

[B34a] Walter X. A. (2011). Anaerobic Iron Cycling in a Neoarchean Ocean Analogue. Ph.D. thesis, University of Neuchâtel, Switzerland. urn:nbn:ch:rero-004-110372

[B35] Widdel F. Schnell S. Heising S. Ehrenreich A. Assmus B. Schink B. (1993). Ferrous iron oxidation by anoxygenic phototrophic bacteria. Nature 362, 834–836 10.1038/362834a0

[B36] Xiong J. Fischer W. M. Inoue K. Nakahara M. Bauer C. E. (2000). Molecular evidence for the early evolution of photosynthesis. Science 289, 1724–1730. 10.1126/science.289.5485.1724 10976061

[B37] Zerkle A. L. Claire M. Domagal-Goldman S. D. Farquhar J. Poulton S. W. (2012). A bistable organic-rich atmosphere on the Neoarchaean Earth. Nat. Geosci. 5, 359–363 10.1038/ngeo1425

